# Complete Genome Sequence of a Rabies Virus Strain Isolated from a Brown Bear (*Ursus arctos*) in Primorsky Krai, Russia (November 2014)

**DOI:** 10.1128/genomeA.00642-16

**Published:** 2016-07-07

**Authors:** Michael Y. Shchelkanov, Andrei A. Deviatkin, Vasily Y. Ananiev, Vladimir G. Dedkov, German A. Shipulin, Nataliya N. Sokol, Irina E. Dombrovskaya, Irina V. Galkina, Mikhail E. Shmelev, Vladimir N. Gorelikov, Valentina N. Kozhan, Marina N. Prosyannikova, Sergei V. Aramilev, Pavel V. Fomenko

**Affiliations:** a Biomedicine School, Far Eastern Federal University, Vladivostok, Primorsky Krai, Russia; b Institute of Biology and Soil Science, Far Eastern Branch of Russian Academy of Sciences, Vladivostok, Primorsky Krai, Russia; c Hygienic and Epidemiological Center in Primorsky Krai, Vladivostok, Primorsky Krai, Russia; d Central Scientific-Research Institute for Epidemiology, Russian Federal Service for Surveillance on Consumer Rights Protection and Human Well-Being, Moscow, Russia; e Inter-Regional Veterinary Laboratory in Primorsky Krai, Ussuriysk, Primorsky Krai, Russia; f Primorsky Branch of Non-Commercial Organization “Amur Tiger,” Vladivostok, Primorsky Krai, Russia; g Amur Branch of “World Wide Fund for Nature,” Vladivostok, Primorsky Krai, Russia

## Abstract

We report here the complete genome sequence (GenBank KP997032) of rabies virus strain RABV/*Ursus arctos*/Russia/Primorye/PO-01/2014, isolated in November 2014 from a brown bear (*Ursus arctos*) that attacked a person in Primorsky Krai (Russian Federation). This strain was clustered into the Eurasian genetic subgroup of genotype 1 (street rage).

## GENOME ANNOUNCEMENT

Rabies rhabdovirus (RABV) (prototype), Aravan virus (ARAV), West Caucasian bat virus (WCBV), Irkut virus (IRKV), and Khujand virus (KHUV), which belong to the genus *Lyssavirus* (order *Mononegavirales*, family *Rhabdoviridae*), cause deadly acute encephalitis (rabies) in humans and animals. RABV is clustered nowadays into 7 genotypes: 1, street rage; 2, Lagos bat virus (LBV); 3, Mokola virus (MOKV); 4, Duvenhage virus (DUVV); 5, European bat lyssavirus type 1 (EBLV 1); 6, European bat lyssavirus type 2 (EBLV 2); and 7, Australian bat lyssavirus (ABLV) (1).

RABV and IRKV are circulating in natural biocenoses in the Russian Far East, the northeastern provinces of China, and the Korean peninsula (1–3). However, the ecology of lyssaviruses in this region needs more detailed investigation.

An attack of a brown bear (*Ursus arctos*) on a person was registered in November 2014 in the village of Barabash (Khasan region of Primorsky Krai), which is located in close proximity to the “Land of the Leopard” national park. The bear was killed, and the presence of RABV antigens in the bear’s brain was confirmed using fluorescence immune assay (FIA) and enzyme-linked immunosorbent assay (ELISA). Strain RABV/*Ursus arctos*/Russia/Primorye/PO-01/2014 (henceforth, PO-01) was isolated from the bear’s brain following the model used for intracerebral inoculation of newborn mice and was identified with the help of FIA, ELISA, reverse transcription PCR, and complete genome sequencing using the primer set described previously (4).

PO-01 is the first completely sequenced Far Eastern strain of RABV and can be considered as topotypic. PO-01 considerably differs from the vaccine strain RV-97 (GenBank accession no. EF542830), which was the basis for the attenuated vaccine applied to the “Land of the Leopard”: 5.6% difference between nucleotide sequences of the N gene (2.7% between amino acid sequences); P gene, 9.3% (9.4%); M gene, 9.2% (7.4%); G gene, 9.5% (9.5%); and L gene, 7.1% (2.8%). At the same time, the immunodominant sites in the PO-01 and RV-97 proteins differ slightly, and the application of the vaccine strain can be recommended for continuation.

The analysis of the N and G genes of PO-01 revealed that it belongs to the Eurasian genetic subgroup of genotype 1 (street rage). Therefore, this genetic subgroup stretches to the east up to the suburb of the continent. Thus, three genetic subgroups of RABV could circulate simultaneously in the Russian Far East: the so-called Arctic (Daur-Manchurian line), East Chinese, and street rage subgroups. The expansion of the cross-border protected territories of the Russian Federation and China in the Far East demands the correct accounting of the circulation of lyssaviruses, which could pose threats for humans and especially for valuable species of animals (1, 3–5). 

### Nucleotide sequence accession number.

The genome sequence of strain RABV/*Ursus arctos*/Russia/Primorye/PO-01/2014 has been deposited in GenBank under the accession number KP997032.
